# Installation Planning in Regional Thermal Power Industry for Emissions Reduction Based on an Emissions Inventory

**DOI:** 10.3390/ijerph16060938

**Published:** 2019-03-15

**Authors:** Yu Zhang, Jiayu Wu, Chunyao Zhou, Qingyu Zhang

**Affiliations:** 1 Department of Environmental Engineering, Zhejiang University, Hangzhou 310058, China; zy_zhangyu@zju.edu.cn (Y.Z.); mouciren@zju.edu.cn (C.Z.); 2 Appraisal Center for Environment and Engineering, Ministry of Environmental Protection, Beijing 100012, China; wujy@acee.org.cn

**Keywords:** thermal power industry, air pollution, emissions inventory, installation planning

## Abstract

Exploring suitable strategies for air pollution control, while still maintaining sustainable development of the thermal power industry, is significant for the improvement of environmental quality and public health. This study aimed to establish a coupling relationship between installed capacity versus energy consumption and pollutant emissions, namely the installed efficiency, and to further provide ideas and methods for the control of regional air pollutants and installation planning. An inventory of 338 installed thermal power units in the Jing-Jin-Ji Region in 2013 was established as a case study, and comparisons were made by clustering classification based on the installed efficiencies of energy consumption and pollutant emissions. The results show that the thermal power units were divided into five classes by their installed capacity: 0–50, 50–200, 200–350, 350–600, and 600+ MW. Under the energy conservation and emissions reduction scenario, with the total installed capacity and the power generation generally kept constant, the coal consumption was reduced by 17.1 million tons (8.7%), and the total emissions were reduced by 79.8% (SO_2_), 84.9% (NO_x_), 60.9% (PM), and 59.5% (PM_2.5_).

## 1. Introduction

Currently, thermal power is still an indispensable part of the electric power industry. The world’s total thermal power generation was 9.41 × 10^11^ GWh in 2015, which will continue to increase in the following 10 years and is expected to reach1.01 × 10^12^ GWh in 2025 [1]. However, air pollution from thermal power industry is an important cause of haze, being responsible for 35% of haze in China [2], with an annual 1% increase exposure to fine particulate matter (PM_2.5_) corresponding to a 2.942% increase in household health expenditure [3]. Previous studies have stated that the introduction of air control policies can help with the improvement of environment quality and public health [4,5]. Therefore, exploring suitable strategies for air pollution control, while maintaining sustainable development of the thermal power industry, is currently one of the issues receiving attention.

It has been shown that, in some industries, air pollutant emissions from 30% of pollution sources with the most outdated processes and technology account for 58%–82% of the industry’s total [6]. To determine the key areas to control emissions, and ultimately provide a solution to pollution strategies and installation planning, it is prudent to explore whether the aspects of low production capacity and air pollution also prevail in the thermal power sector.

To date, many emission inventories of pollutant sources have been established and studied to develop emissions reduction strategies in China and other countries [7,8,9,10]. However, these inventories analyzed the emissions reduction strategies based only on the level of emissions; even if the production scale was taken into account, no relationship was established between pollutant emissions and production scale [9]. Hence, after an emissions inventory is established, it is still necessary to establish a coupling relationship between production capacity and pollutant emissions, determine priorities for emissions reduction, and propose a reasonable emissions reduction strategy.

The Lorenz curve is generally used to illustrate the distribution of national income among a country’s population, and its function is to visualize the unequal relation of two variables [10]. In recent years, the types of variables illustrated by the Lorenz curve have become more extensive. This curve has been used to establish unequal relations between socio-economic variables, such as population, income, and gross domestic product, versus pollutant emissions, as well as in environmental fields, such as carbon emissions reduction [11,12,13]. The Gini coefficient can comprehensively examine the degree of distribution equality, because the curvature of the Lorenz curve is proportional to the Gini coefficient. An emissions reduction strategy may create inequality if it is based solely on the level of pollutant emissions without taking into account the impact of equipment and technology. Thus, the Lorenz curve can be used to analyze emissions reduction strategies in the thermal power industry by considering equipment, technology, and energy consumption, while the Gini coefficient can be adopted to estimate the inequality index of emissions from thermal power units. K-means clustering is a typical partition clustering algorithm that has been applied to determine the air index, and the association between air pollution and human health [14,15,16]. Owing to its simplicity and efficiency, k-means clustering is suitable for rapid grouping under the impact of multiple factors. Because the effects of three factors, namely installed capacity, coal consumption, and various pollutant emissions, should be taken into account for emissions reduction strategies in the thermal power industry, k-means clustering enables rapid grouping of installations according to the pollutant emissions inventory.

This paper aims to establish a coupling relationship between installed capacity versus energy consumption and pollutant emissions, and to further provide ideas and methods for the control of regional air pollutants and installation planning. The thermal power industry in the Beijing-Tianjin-Hebei (Jing-Jin-Ji) Region was selected for case analysis of SO_2_, NO_x_, and particulate matter (PM) emissions in the year 2013. We established an inventory of the production scale, coal consumption, and air pollutant emissions, and determined the inequality by using the Lorenz curve. A coupling relationship between installed capacity versus energy consumption and pollutant emissions was then established, namely the installed efficiency. Further, we used k-means clustering to classify thermal power units by their installation scale, analyzed the emission characteristics, and devised an energy conservation and emissions reduction (EE) scenario, thereby determining industry shutdown limits, entry thresholds, and industry upgrades based on the efficiency of pollutant emissions

## 2. Methods

### 2.1. Data Collection

The geographic location, unit information, coal consumption, flue gas emissions, pollutant control, and stack parameters of thermal power units in the Jing-Jin-Ji Region were collected and improved through the cooperation of the Environmental Engineering Assessment Center of the Ministry of Environmental Protection, local environmental protection departments, and the power generation groups. In addition, the database was checked and supplemented by a combination of field investigation and questionnaires. The details are shown in Table 1.

### 2.2. Establishment and Verification of Emissions Inventory

The emissions inventory was established based on pollution source survey methods to collect basic information, such as exact location, size, coal consumption, and power generation hours of coal-fired power-generating units in the Jing-Jin-Ji Region. The emissions factor method was used to calculate the emissions and emission rates from coal-fired units according to the mean hourly concentration of specific pollutant emissions, flue gas volume under standard conditions, and power generation hours during the study year. The pollutant emissions of SO_2_, NO_x_, and PM were calculated as follows:

(1) $$Q=\sum_{i=1}^{St}C_{i}\times L_{i}\times 10^{-6}$$

where *Q* is the annual emission of a pollutant in kilograms per hour, *C_i_* is the mass concentration of hourly emissions of dry flue gas under standard conditions in milligrams per cubic meter, *L_i_* is the dry flue gas volume under standard conditions in cubic meters per hour (here, *L_i_* is estimated based on the coal consumption of 8.5–9 m^3^/kg according to the installation class), and *St* is the power generation hours within one year.

Because PM_2.5_ monitoring was not specifically performed, PM_2.5_ emissions were quantified based on their mass ratio in PM (the ratio gradually increased with decreasing PM concentration) to derive final emissions values. 

According to the practical investigation, the actual total installed capacity of the thermal power industry in the Jing-Jin-Ji Region was 54,801 MW. This result was 2% lower than that recorded in the statistical yearbook, mainly because some enterprises had no production.

According to the investigation and verification, the total emissions from thermal power units in this region were 298,431.18 (SO_2_), 60,321.190 (NO_x_), and 72,007.47 (PM) tons, while the values were 311,706.7 (SO_2_), 709,217.7 (NO_x_), and 85,140.7 (PM) tons, according to the Annual Statistic Report [17]. The probable reason is that the statistics of some thermal power plants in the region were not accurately recorded.

### 2.3. Establishment of Unequal Relations

To analyze the inequality of pollutant emissions from different scales of thermal power units, the units were ranked by size in descending order. We calculated the ratio of cumulative capacity *Y*(*i*) and the cumulative ratio *X*(*i*) of SO_2_, NO_x_, PM, and PM_2.5_ emissions. A Lorenz curve for installed capacity versus pollutant emissions was drawn according to the function

(2) Y=L(X)

The Gini coefficient (*G*) was calculated by the curve fitting method using a polynomial function:

(3) $$\hat{Y}=\hat{L}\left(X\right)=a_{n}X^{n}+a_{n-1}X^{n-1}+\ldots +a_{1}X+a_{0}$$

where *n* = 6. The value of *G* was calculated using the following formula of integration:

(4) $$G=1-2\int_{0}^{1}L\left(X\right)dX\approx 1-2\int_{0}^{1}\hat{L}\left(X\right)dX$$

### 2.4. Installed Efficiency

To establish the relationship between production scale versus pollutant emissions and coal consumption, we calculated the SO_2_, NO_x_, PM, and PM_2.5_ emissions and coal consumption per unit installed capacity, that is, the installed efficiency:

(5) $$E_{ij}=\frac{P_{ij}}{C_{ij}}$$

where *E_ij_* is the installed efficiency of thermal power units with installed capacity *I* in the range of *i–j* (*i* ≤ *I* < *j*); *C_ij_* is the total installed capacity of thermal power units with installed capacity in the range of *i–j*; and *P_ij_* is the annual emissions of SO_2_, NO_x_, PM, and PM_2.5_ and coal consumption in thermal power units with installed capacity in the range of *i–j*.

### 2.5. K-Means Clustering Analysis

The SPSS statistical analysis software (IBM SPSS, Somers, NY, USA) was used to perform k-means clustering for thermal power enterprises classified into different installation scales. The variable for installed efficiency was calculated at 50 MW intervals. The total installed capacity of units with 400–450, 450–500, and 550–600 MW of thermal power was 0, and thus their installed efficiency was also 0, and these sizes were not included in the clustering process. The number of clusters was determined using the elbow method. The maximum number of iterations was 10 and the convergence criterion was 0.02.

### 2.6. Case Study

**Figure 1:**
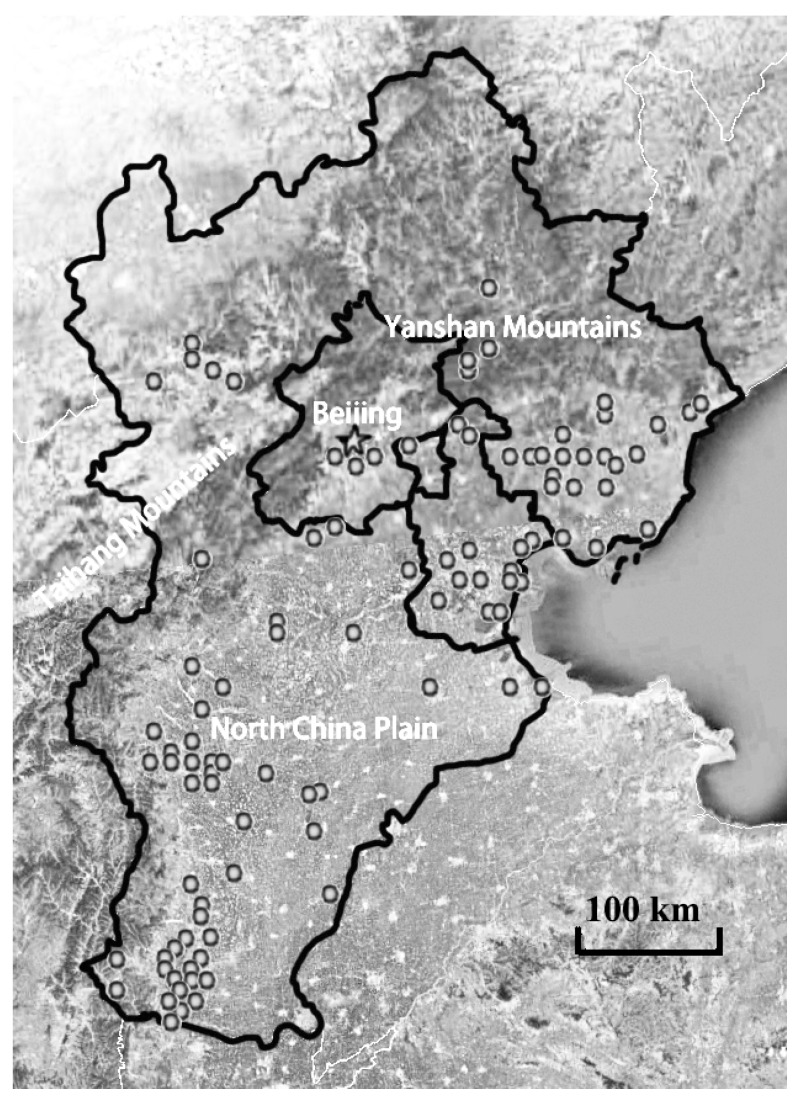
Distribution of thermal power units in the Jing-Jin-Ji Region.

The Jing-Jin-Ji Region is located in the western part of China’s Bohai Economic Rim, at the northern end of the North China Plain. In this region, there are 11 prefecture-level cities, including Beijing, Tianjin, and Hebei, and it has a total area of 21.80 × 10^4^ km^2^. This region has experienced one of the fastest economic developments in the world over the past 30 years, and it is also one of the regions with the most serious air pollution in China. In January 2013, the Jing-Jin-Ji Region experienced the worst persistent haze pollution incident in history [18]. That same year, the Ministry of Environmental Protection of China announced the top 10 cities with most serious air pollution [19], 8 of which are in the Jing-Jin-Ji Region. Thermal power units in this region are mainly concentrated in the North China Plain where terrain is flat, especially in the southern area and the eastern coast of this region (Figure 1).

The Jing-Jin-Ji Region has numerous installations for China’s thermal power industry. According to the statistical yearbook [20], the thermal power installed capacity in Jing-Jin-Ji Region was 59.70 GW in 2013, that is, 6.9% of the country’s installed thermal power capacity; yet this only accounts for 2.3% of the country’s total area. From 2005 to 2013, thermal power installed capacity grew by 84.6% and thermal power generation grew by 66.8% in the Jing-Jin-Ji Region.

As the thermal power industry is the main coal-consuming industry in the Jing-Jin-Ji Region, its pollutant emissions have a profound impact on regional air quality. A recent study [21] showed that emissions from thermal power industry in the Jing-Jin-Ji Region accounted for 25.02% (SO_2_), 39.55% (NO_x_), and 5.73% (PM_10_, inhalable particles, <10 μm) of the region’s total emissions of these pollutants. According to the Environmental Quality Bulletin of the Beijing-Tianjin-Hebei Region [22,23,24], in 2013, the mean annual concentrations of pollutants such as NO_2_, PM_2.5_ (fine particles, <2.5 μm), and PM_10_ (excluding SO_2_) exceeded the national standards in Beijing and Tianjin, while the mean annual concentrations of all pollutants exceeded the national standards in Hebei Province. Thus, it is representative to select the thermal power industry in the Jing-Jin-Ji Region for case analysis in the year 2013.

## 3. Results and Discussion

### 3.1. Production Scale and Emissions Data

**Figure 2:**
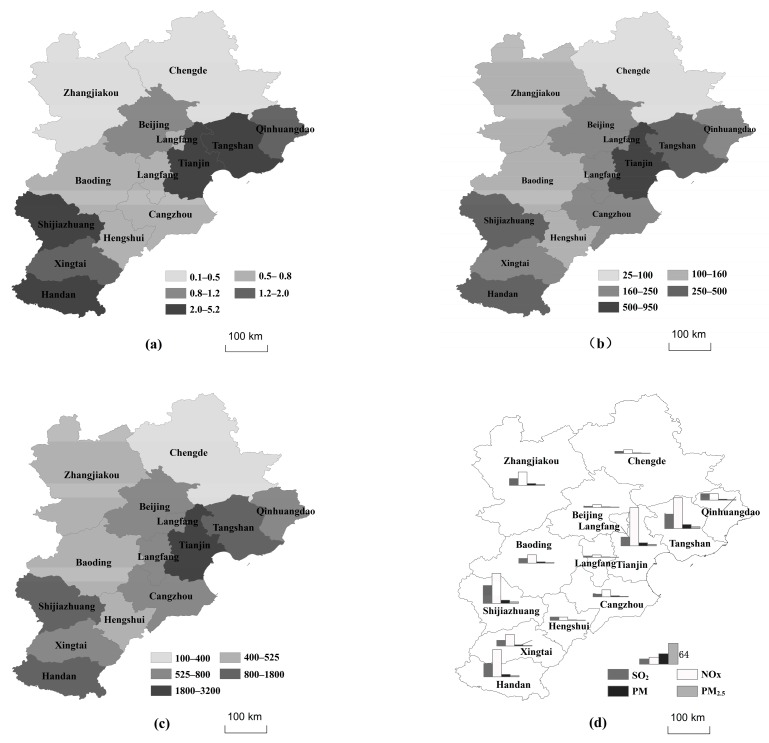
Spatial distribution of thermal power units in the Jing-Jin-Ji Region: (**a**) installations per unit area (10^−3^ units/km^2^), (**b**) installed capacity per unit area (kW/km^2^), (**c**) coal consumption per unit area (ton/km^2^/year), (**d**) annual emissions (10^−3^ ton/year).

There were 133 thermal power enterprises (groups) in the Jing-Jin-Ji Region in 2013, with a total of 338 installed units and a total installed capacity of 54,800.5 MW. The number of installed thermal power units per unit area and the installed capacity in different cities are detailed in Figure 2a,b. The installed capacity of thermal power units in Tianjin, Tangshan, Xingtai, and Handan was far greater than that in other cities. As is shown in Figure 1, these areas have flat terrain with more plains than mountains, and therefore are suitable for the construction of thermal power units. 

The total coal consumption of the thermal power industry in the Jing-Jin-Ji Region was 196.3914 million tons, accounting for 10.34% of the total thermal coal consumption in China [20]. The spatial distribution of thermal power coal consumption per unit area, as shown in Figure 2c, was consistent with that of the installed level in the Jing-Jin-Ji Region. The total emissions of the thermal power industry in Jing-Jin-Ji Region were 146,791.87 (SO_2_), 351,303.49 (NO_x_), 36,712.58 (PM), and 18,551.32 (PM_2.5_) tons. The pollutant emissions of different cities are shown in Figure 2d.

### 3.2. Unit Classification 

**Figure 3:**
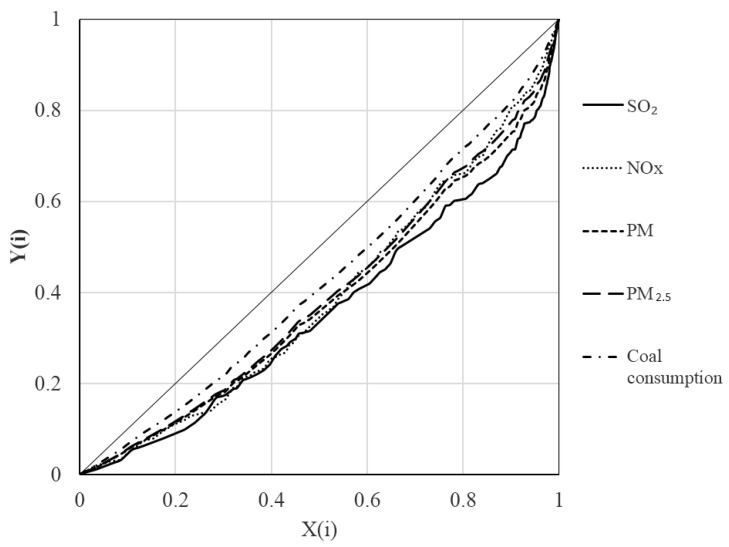
Lorenz curve of thermal power units in Jing-Jin-Ji Region.

The Lorenz curve of pollutant emissions in Jing-Jin-Ji Region is shown in Figure 3. The emissions of thermal power units with *Y*(*i*) > 80% accounted for 39.7% (SO_2_), 34.0% (NO_x_), 34.7% (PM), and 32.8% (PM_2.5_) of the total emissions. That is, the SO_2_ emissions from the smallest 20% of units contributed to 39.7% of the total emissions.

By using the curve fitting method, the Gini coefficients of emissions were calculated to be 0.28 (SO_2_), 0.22 (NO_x_), 0.23 (PM), and 0.21 (PM_2.5_), showing a significant inequality of emissions. Hence, the thermal power units needed to be classified to study pollutant emissions and emissions reduction strategies for different classes.

The elbow method determined the number of clusters to be 5, and then k-means clustering was performed. Results are given in Table 2. Because no units had an installed capacity of 400–450, 450–500, or 550–600 MW thermal power, the fourth class was combined into the 350 to 600 MW class. In this way, the thermal power units were divided into five classes by their installation scale: 0–50, 50–200, 200–350, 350–600, and 600+ MW.

### 3.3. Emission Characteristics

**Figure 4:**
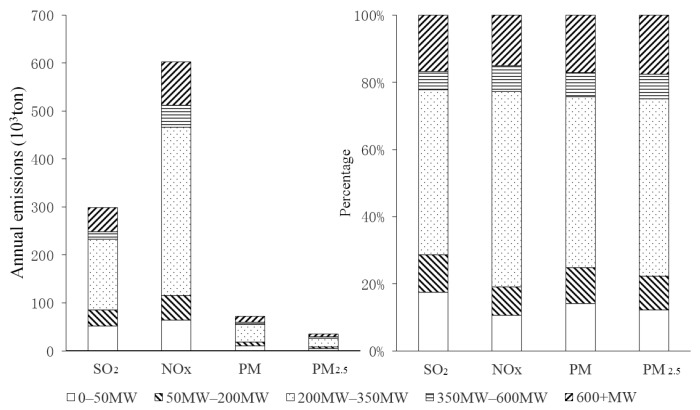
Pollutant emissions and ratios for each class.

The pollutant emissions and their ratio for each class of units are shown in Figure 4. The largest pollutant emissions level was found in thermal power units of 200–350 MW, where the total emissions were 146,791.87 (SO_2_), 351,303.49 (NO_x_), 36,712.58 (PM), and 18,551.32 (PM_2.5_) tons, with corresponding emission ratios of 49.2% (SO_2_), 58.2% (NO_x_), 51.0% (PM), and 52.9% (PM_2.5_). The installed capacity of this class of units accounted for 53.1% of the total installed capacity, which was comparable to the emission ratios. In addition, the installed capacity of 0 to 50 MW units accounted for 3.4% of the installed capacity, and their total emissions were 52,060.57 (SO_2_), 63,837.98 (NO_x_), 10,219.44 (PM), and 4306.38 (PM_2.5_) tons, with corresponding emission ratios of 17.4%, 10.6%, 14.2%, and 12.3%. According to these results, the effect of emissions reduction by shutting down units <50 MW was significant.

### 3.4. Installed Efficiency

**Figure 5:**
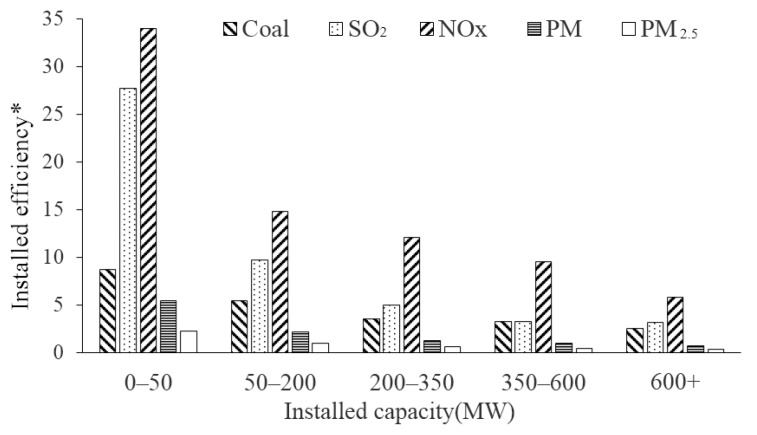
Installed capacity. * The unit of installed efficiency for coal consumption is 10^3^ ton/MW, and the unit of installed efficiency for pollutants is ton/MW.

The installed efficiency of different classes of thermal power units is shown in Figure 5. There were marked differences in the efficiency of pollutant emissions and coal consumption among the various classes. In other words, when converted to the same scale of power generation capacity, the coal consumption and pollutant emissions of small units were much greater than those of large units. For 0–50 MW of installed capacity, the installed efficiency reached 27.76 (SO_2_), 34.04 (NO_x_), 5.45 (PM), and 2.30 (PM_2.5_) kilotons/MW. With 600+ MW installed capacity, the installed efficiency was only 3.21 (SO_2_), 5.83 (PM), 0.80 (PM), and 0.40 (PM_2.5_) kilotons/MW.

**Figure 6:**
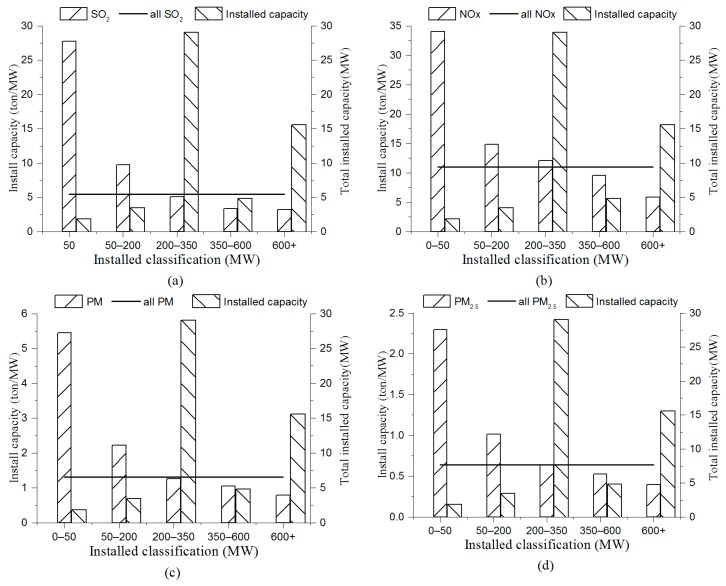

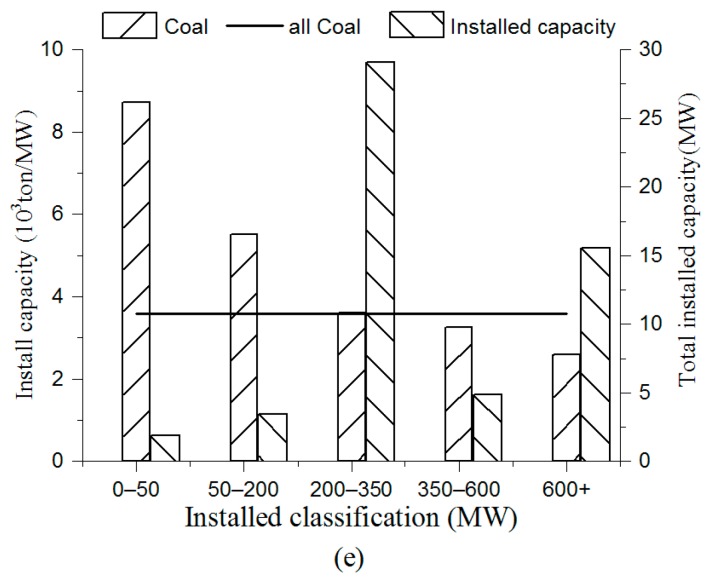
Installed capacity and installed efficiency: (**a**) installed capacity and SO_2_ emissions installed efficiency, (**b**) installed capacity and NO_x_ emissions installed efficiency, (**c**) installed capacity and PM emissions installed efficiency, (**d**) installed capacity and PM_2.5_ emissions installed efficiency, (**e**) installed capacity and coal consumption installed efficiency.

The relationships between the installed efficiency of different classes versus the total installed capacity and mean installed efficiency are illustrated in Figure 6. By comparing the installed efficiency and the total installed capacity of different classes, it was derived that the units with 200–350 MW thermal power had the highest total installed capacity, while their installed efficiency of pollutant emissions and coal consumption was comparable to the average level and ranked third. Therefore, reducing the overall installed efficiency of 200–350 MW through a process improvement approach would significantly save energy and reduce emissions. The remaining four classes were units with 600+, 350–600, 50–200, and 0–50 MWh, in descending order according to the total installed capacity. Their corresponding installed efficiency rankings were fourth, fifth, second, and first. It is necessary to limit the production of units with 0–350 MW and encourage the construction of new units with 600+ MW. By comparing the installed efficiency and the mean installed efficiency of all classes, it was derived that the installed efficiency of pollutant emissions and coal consumption in thermal power units with 350+ MW was below the average level. The probable reason is that technologies of the thermal power units with 0–350 MW are limited and backward. Thus, it is more conducive to achieving the EE goals to restrict the installed capacity of new thermal power units to no less than 350 MW.

### 3.5. Energy Conservation and Emissions Reduction Scenario

An EE scenario was established according to the above analysis, combined with related standard bulletins in the thermal power industry [25,26,27,28,29], and compared with a “business as usual” (BAU) scenario for the thermal power industry in the Jing-Jin-Ji Region in 2013. We assumed that the coal quality was constant and that the total power generation and total installed capacity were generally kept constant. The EE scenario had the following characteristics:

(1) All <50 MW units were shut down.
(2) The 50–200 MW units met special emission standards (with emission concentrations no greater than 50 (SO_2_), 100 (NO_x_), and 20 (PM) mg/m^3^), with a standard coal consumption for power generation that was no greater than 293 g/kWh.
(3) The 200–350 MW thermal power units met ultra-low emission standards (with emission concentrations no greater than 35 (SO_2_), 50 (NO_x_), and 10 (PM) mg/m^3^), with a standard coal consumption for power generation that was no greater than 293 g/kWh.
(4) The existing 350–600 MW and 600+ MW thermal power units met ultra-low emission standards (as given in item 3), with a standard coal consumption for power generation no greater than 293 and 284 g/kWh when installed capacity was increased by 5% and 10%, respectively. The newly installed units met ultra-low emission standards and the standard coal consumption for power generation by the new thermal power units was no greater than 287 and 270 g/kWh, respectively.

The production scale, annual (a) coal consumption, and annual pollutant emissions of different unit classes under the BAU and EE scenarios are shown in Table 3.

The results show that under the EE scenario, different unit classes had the following effects on the total production scale and total pollutant emissions with respect to the BAU scenario:

(1) Shutting down the 0–50 MW thermal power units reduced the installed capacity, power generation, and coal consumption by 3.4%, 3.7%, and 8.3%, respectively; the total emissions were reduced by 17.4% (SO_2_), 10.6% (NO_x_), 14.2% (PM) and 12.3% (PM_2.5_).
(2) For the 50–200 MW units, the EE scenario with constant installed capacity and power generation reduced coal consumption by 0.9%; the total emissions by 8.6% (SO_2_), 5.8% (NO_x_), 5.9% (PM), and 5.1% (PM_2.5_).
(3) For the 200–350 MW units, the EE scenario with constant installed capacity and power generation reduced coal consumption by 1.9%; the total emissions were reduced by 38.1% (SO_2_), 50.4% (NO_x_), 24.7% (PM), and 25.9% (PM_2.5_).
(4) For the 350–600 MW units, the EE scenario increased the installed capacity, power generation, and coal consumption by 0.4%, 0.4%, and 0.4%, respectively; the total emissions were reduced by 3.6% (SO_2_), 6.4% (NO_x_), 5.0% (PM), and 5.1% (PM_2.5_).
(5) For the 600+ MW units, the EE scenario increased the installed capacity, power generation, and coal consumption by 2.8%, 2.8%, and 2.1%, respectively; the total emissions were reduced by 12.1% (SO_2_), 11.7% (NO_x_), 11.1% (PM), and 11.4% (PM_2.5_).

The EE scenario considerably reduced pollutant emissions and saved more energy compared with the BAU scenario. Table 4 shows that when the total installed capacity and power generation were generally constant, the coal consumption was reduced by 17.1 million tons (8.7%), and the total emissions were reduced by 79.8% (SO_2_), 84.9% (NO_x_), 60.9% (PM), and 59.5% (PM_2.5_). Compared with a previous study [30], the total installed capacity of thermal power units increased by 4.1%, while total emissions were reduced by 15.0% (SO_2_), 12.6% (NO_x_), and 8.4% (PM).

## 4. Conclusions

In this study, an inventory of production scale, coal consumption, and air pollutant emissions was used to provide a reference for an energy conservation and emissions reduction strategy in the thermal power industry. Comparisons were made by clustering classification based on the installed efficiencies of energy consumption and pollutant emissions. This method can also apply to other polluting industries for the analysis of EE strategies from the perspective of emissions inventory.

Our results showed that emissions from the smallest 20% of thermal power units accounted for 39.7% (SO_2_), 34.0% (NO_x_), 34.7% (PM), and 32.8% (PM_2.5_) of total emissions from thermal power units in the Jing-Jin-Ji Region in 2013. According to the installed efficiency, the thermal power units were divided into five classes by their installation scale: 0–50, 50–200, 200–350, 350–600, and 600–1,000 MW. The analysis of emission characteristics revealed that units <50 MW had low total installed capacity and a high ratio of pollutant emissions, and we recommend that these plants be shut down as soon as possible. The analysis of installed efficiency revealed that units >350 MW had installed efficiency of energy consumption and pollutant emissions below the average level, so we further recommend that installed capacity of new thermal power units be restricted to no less than 350 MW.

Compared with BAU, the EE scenario not only considerably reduced the pollutant emissions of thermal power units, but also saved energy: when the total installed capacity and power generation were generally constant, the total coal consumption was reduced by 17.1 million tons (8.7%), whereas the total emissions were lowered by 79.8% (SO_2_), 84.9% (NO_x_), 60.9% (PM), and 59.5% (PM_2.5_).

## Figures and Tables

**Table 1:** Information gathering methods.

Information Category	Specific Description
Geographic location	City, province and longitude, latitude (°/′/′′)
Unit information	Unit number, installation thermal capacity, boiler tonnage, construction conditions, construction property, and power generation hours
Coal consumption construction	Standard coal consumption for power generation, coal consumption, coal quality information (ash content, sulfur content, nitrogen content, mercury content, coal source, and calorific value)
Flue gas emissions	Flue gas volume, SO_2_ concentration, SO_2_ emission rate, NO_x_ concentration, NO_x_ emission rate, PM concentration, and PM emission rate
Pollutant control	Treatment process, treatment efficiency, emission concentration, and emission load
Chimney parameters	Height, outlet diameter, outlet temperature, flow rate, and emission pattern

**Table 2:** K-means clustering of installed efficiency.

Installed Capacity	Coal Consumption (10^3^ ton/MW)	SO_2_ (ton/MW)	NO_x_ (ton/MW)	PM (ton/MW)	PM_2.5_ (ton/MW)	Cluster
0–50	0.87	27.76	34.04	5.45	2.3	1
50–100	0.58	10.3	15.07	2.63	1.18	2
100–150	0.54	9.92	13.1	1.78	0.87	2
150–200	0.53	10.05	14.69	2.45	1.06	2
200–250	0.46	4.94	13.8	1.16	0.6	5
250–300	0.4	4.84	12.33	1.17	0.62	5
300–350	0.36	5.13	11.82	1.29	0.65	5
350–400	0.31	3.58	11.71	1.05	0.53	4
400–450 *	-	-	-	-	-	-
450–500 *	-	-	-	-	-	-
500–550	0.35	2.93	12.08	1.05	0.52	4
550–600 *	-	-	-	-	-	-
600–650	0.27	3.54	6	0.83	0.42	3
650–700	0.26	3.18	5.28	0.81	0.4	3
1000+	0.21	1.71	6.09	0.57	0.29	3

* The total installed capacity is 0 in 400–450, 450–500, and 550–600 MW, so the installed efficiency does not exist.

**Table 3:** Scenario analysis results.

Installed Classification (MW)	Scenario	Number of Installed Units	Installed Capacity (MW)	Power Generation (10^6^ MWh)	Coal Consumption (10^6^ ton/a)	SO_2_ (10^3^ ton/a)	NO_x_ (10^3^ ton/a)	PM (10^3^ ton/a)	PM_2.5_ (10^3^ ton/a)
0–50	BAU	156	1875.5	12.2	16.4	52.1	63.8	10.2	4.3
	EE	0	0	0	0	0	0	0	0
50–200	BAU	41	3453.0	21.6	19.0	33.6	51.3	7.7	3.5
	EE	41	3453.0	21.6	17.2	7.9	16.3	3.4	1.7
200–350	BAU	105	29,082.0	174.3	105.0	146.8	351.3	36.7	18.6
	EE	105	29,082.0	174.3	101.2	33.1	47.3	18.9	9.5
350–600	BAU	12	4830.0	27.9	15.7	16.0	46.1	5.1	2.5
	EE	12	5071.5	29.3	16.5	5.2	7.4	1.5	0.7
600+	BAU	24	15,560.0	94.0	40.3	50.0	90.7	12.4	6.2
	EE	25	17,116.0	103.4	44.3	14.0	19.9	4.3	2.2

BAU: business as usual; EE: energy-conservation and emissions-reduction. Ton/a: ton/annual.

**Table 4:** Scenario comparison.

Scenario Comparison	Installed Capacity (MW)	Power Generation (10^6^ MWh)	Coal Consumption (10^6^ ton/a)	SO_2_ (10^3^ ton/a)	NO_x_ (10^3^ ton/a)	PM (10^3^ ton/a)	PM_2.5_ (10^3^ ton/a)
BAU	54,800.5	330.1	196.4	298.4	603.2	72.0	35.1
EE	54,722.5	328.7	179.3	60.2	90.9	28.1	14.1
Ratio	−0.1%	−0.4%	−8.7%	−79.8%	−84.9%	−60.9%	−59.9%
